# Literary Notes

**Published:** 1909-08

**Authors:** 


### LITERARY NOTES

---

The J. B. Lippincott Company, Philadelphia and London, announce the early publication of a handbook of Medical Diagnosis, by J. C. Wilson, A. M., M. D., Professor of the Practice of Medicine and Clinical Medicine in the Jefferson Medical College, and Physician to its Hospital; Physician to the Pennsylvania Hospital; Physician in Chief to the German Hospital, Philadelphia, the same to contain more than 300 illustrations.

---

The W. B. Saunders Company has issued a new revised edition of their illustrated catalogue. For this edition it has been subjected to a most thorough revision, incorporating some twenty-five new books and new editions. The colored plate from Keen's new Surgery, replacing the one inserted in the last edition, and the colored illustration of the spirochaeta pallida as stained by the method of Levaditi,—illustrating the announcement of Jordan's general bacteriology,—in themselves give the catalogue a real value. Printed in two colors on a high grade India tint paper and handsomely bound, this edition is truly an edition de luxe.

---

THE *Annals of Surgery* for July, 1909, is in some respects, the most remarkable magazine relating to medicine and surgery that has been published in this country. It consists of 366 standard octavo pages, given to the publication of the papers read at the meeting of the American Surgical Association, held at Philadelphia, June 3, 4, and 5, 1909, together with the proceedings, the latter including abstracts of the discussions. Numerous illustrations are placed in the text of the papers, and some plates are introduced. On the whole, it is a remarkable edition of a splendid medical periodical, a credit to editors and publishers, and deserves the praise of the medical profession the world over.

